# Access to physiotherapy services in South African settings: stretching a hand to under-resourced communities with students as agents of change

**DOI:** 10.3389/fresc.2023.1164525

**Published:** 2023-07-27

**Authors:** Kganetso Sekome, Laeeqa Sujee, Lauren Tomes, Sonti Pilusa

**Affiliations:** Department of Physiotherapy, Faculty of Health Sciences, University of the Witwatersrand, Johannesburg, South Africa

**Keywords:** physiotherapy, access, advocacy, diversity & inclusion, equity

## Abstract

**Background:**

Physiotherapy is a healthcare profession that enhances human functioning and prevents further disability. In addition to this physiotherapy promotes health, wellbeing and the prevention of diseases. In South Africa, physiotherapy and rehabilitation services are largely accessed by those living in urban communities and those with private medical funding. Rehabilitation is an essential component of the package of care yet these services do not reach disadvantaged communities who vitally need them. Through the equitable provision of service-learning, physiotherapy students can play a vital role in improving rehabilitation access to vulnerable communities.

**Aim:**

This paper illustrates how physiotherapy students studying at a South African university provide equitable services to disadvantaged rural and urban communities.

**Discussion:**

The first and second years of study focus on theoretical and classroom-based learning where students gain an understanding of basic principles of inclusion, equity, diversity, and self-awareness. In later years, students provide community-based services in urban and rural communities with a focus on increasing access to rehabilitation services. The clinical objectives which students are required to fulfil are guided by principles of public health and community rehabilitation. The Covid-19 pandemic highlighted the issue of poor access to rehabilitation services and the case study we provide demonstrates the roles physiotherapy students played to fill this gap. The authors offer a reflection from their involvement in physiotherapy student training and provide an example of a moment which displayed equity, diversity, and inclusion in their career.

**Conclusion:**

There is a huge gap to fill in the provision of equitable rehabilitation services for the South African population. Institutions responsible for the training of physiotherapists, or any rehabilitation profession, should realize their role in service delivery through a service-learning approach.

## Background

The history of the South African health system is well documented with multiple policies dating back to pre-apartheid colonial subjugation. The vast income inequalities (1), a two-tiered health care system consisting of public and private sectors (2), and geographical sub-divisions have resulted in public health challenges where those who are rich or well-geographically positioned are favoured. A lack of human resources in the public health sector (3, 4), coupled with poor management has resulted in an inadequate response to the basic health needs of the country. People with disabilities and those who lack access to basic health services have been historically disadvantaged and left with poorer health outcomes (5), despite the well documented South African policies acknowledging their needs. These are policies that are infamously known to be poorly implemented, especially considering the diverse needs of the two-tiered system.

The private health sector caters to <20% of the South African population via medical insurance, out-of-pocket payments, and hospital plans, while the vulnerable >80% population relies on the public health sector (2, 5). Public health service users are often deprived access to rehabilitation services due to factors such as long distances to facilities, maldistribution and lack of rehabilitation health workers, lack of assistive devices to enable mobility and transportation to the facility, or simply not knowing about rehabilitation and what it offers (5–7). The need for rehabilitation services in South Africa has been highlighted with the shift from communicable to non-communicable diseases (6, 8) which has placed a huge demand on the provision of rehabilitation services.

Rehabilitation has not been a priority in the South African health sector, instead, there has been a huge focus on saving lives from communicable diseases which has proven successful (5, 9). However, rehabilitation is an essential component of the package of care to ensure universal health coverage across one's life. Rehabilitation is an ongoing process that aims to minimize disability and improve functional ability (10). The components which are central to rehabilitation include preventive care, assessment and intervention using therapeutic approaches and promoting reintegration to the community. The need for rehabilitative care is increasing due to a growing elderly population, sequelae of chronic conditions and musculoskeletal problems (11). The World Health Organization (WHO) launched a global agenda to strengthen rehabilitative care, “Rehabilitation 2030” by improving integration of rehabilitation into the health sector, strengthening multisectoral collaboration, and developing context-specific service delivery models that will ensure access to rehabilitative care (10). Although rehabilitation is an essential component of the package of care, rehabilitation services do not reach disadvantaged communities who need them and therefore strategies are required to strengthen universal health coverage (12).

While the South African government has introduced a reform to the provision of universal health coverage through the National Health Insurance strategy (13), a lot more still needs to be done to address the rehabilitation inequities of the past. The strategy falls short on rehabilitation outcomes (14). These shortfalls include a lack of focus on rehabilitation, a shortage of human resources, and a lack of outreach transportation and assistive devices to name a few.

Training health professionals to respond to the health and rehabilitation needs of marginalised South Africans is one strategy of strengthening universal health coverage. The physiotherapy training programme at the University of the Witwatersrand, South Africa, has a strong focus on empowering students to address issues related to healthcare system access such as diversity, inclusion, and equality. In this paper diversity is conceptualized as an acknowledgment of the differences in terms of race, socioeconomic status, experiences, sexual orientation, ability or disability. In terms of diversity in healthcare, it is the ability of health professionals to respect and render patient-centred care that seeks to understand the patient's needs and context regardless of their race, ethnicity or economic status (15). The concept of diversity is closely linked to inclusion and equity. Inclusion in the rehabilitation setting encompasses patient-centred care where patients or clients are treated according to their specific, individual needs and are encouraged to actively participate in the decision-making process (16). Inclusion and active participation have been identified as essential in addressing the health inequities experienced by people with disabilities (17). This paper illustrates how physiotherapy students training at a South African university practice to address health inequities in disadvantaged rural and urban communities.

## Physiotherapy students as change agents: inclusion, diversity, and equity in practice

The physiotherapy programme at the University of the Witwatersrand is a four year training degree that places emphasis on patient-centred care. The training highlights the importance of understanding the patient and their community's context, professional behaviour and sensitivity to the diverse cultures as well as the importance of applying theories and concepts to address the inequities in health care. The scope of the public health and community physiotherapy curriculum within the physiotherapy training programme exposes students to under-served and under-resourced communities where they must assess the community's health needs and design interventions to address the identified needs.

The structure of the curriculum focuses on aspects of inclusivity, diversity, and equity as outlined in Table 1 and described thereafter. Through this curriculum, it is envisaged that students will go into communities of different contexts and provide physiotherapy and rehabilitation services that addresses the health inequities created partly by apartheid segregation. The curriculum empowers students to be change agents who can make a difference in the diverse context of South Africa. Table 1 outlines the undergraduate curriculum with topics that foster inclusion, diversity and equity.

**Table 1:** Topics covered in the physiotherapy degree programme fostering inclusion, diversity, and equity over a four-year period.

1st year	2nd year	3rd year	4th year
❏ Introduction to diversity and disability ❏ History of physiotherapy ❏ Introduction to public health ❏ Principles of adult learning and health education ❏ Group rehabilitation and group dynamics ❏ Interdisciplinary care ❏ Communication skills ❏ Cultural differences affecting communication ❏ Transformative approach to disability awareness ❏ Cultural, ethnic, religious & educational diversity and their impact on subjective examination ❏ Self-health assessment ❏ Adolescence ❏ Professional behaviour and wellness ❏ International classification of disability, function, and health (ICF) ❏ Bioethics	❏ Health and society ❏ Determinants of health ❏ Understanding health in urban and rural contexts ❏ Health and aging (gerontology) ❏ Rehabilitation process ❏ HIV and rehabilitation ❏ Social exclusion as a determinant of health	❏ Family centred practice ❏ Participation ❏ Childhood disability in the South African context ❏ Integrative medicine ❏ Mental health and awareness ❏ Social security and social grants ❏ Health promotion ❏ Primary health care ❏ Community development ❏ Community based rehabilitation ❏ Home based rehabilitation ❏ Community assessment ❏ Community participation ❏ Ethics in physiotherapy and health care	❏ Health challenges for children in South Africa ❏ Public health laws ❏ Planning and managing physiotherapy services in a district health system ❏ Health screening in a community setting ❏ Occupational health and safety ❏ Self-efficacy and management

### Laying the foundation: first year of study

Students in the physiotherapy programme come from diverse backgrounds of gender, culture, ethnicity, socioeconomic status, and geographical region. The diversity in the student body enables the facilitation of the conversation on the history of physiotherapy which has historically been a white female dominated profession (18). The first year of study is predominantly theoretical learning with case presentations and classroom discussions. The students are taught communication skills as these are central to building a therapeutic relationship with patients and can enhance patient-centred care. The adult learning theory emphasises the need to respect adult learners and to ensure learning is practical and relevant to the needs of adults. This principle is important because when students are in the community they need to render services respectfully and according to the patient or community's needs. Professional behaviour topics include professionalism, reflective practices and wellbeing. Through these topics students come to understand professional behaviour, the importance of reflective practice and personal wellbeing. The students are also introduced to the South African public health system and the contextual challenges that influence health outcomes. These topics become a foundation to developing professional identity, self-awareness and respecting others.

Persons with disability are often marginalised from accessing rehabilitation services, which increases their risk of living in poverty, being unemployed, and having less access to healthcare compared to the general population (19). The first-year students in the physiotherapy programme are introduced to the concept of disability through a transformative approach to disability awareness (20). The topic on disability aims to raise awareness on issues of equity in rehabilitation. The students are given a group activity where they explore challenges facing people with disabilities. This project allows students to better understand the type of everyday issues faced by a person living with a disability. During this project, advocacy is strongly enforced, and the students must discuss how they would advocate for the social inclusion of a person with a disability.

### Second year of study

**Figure 1:**
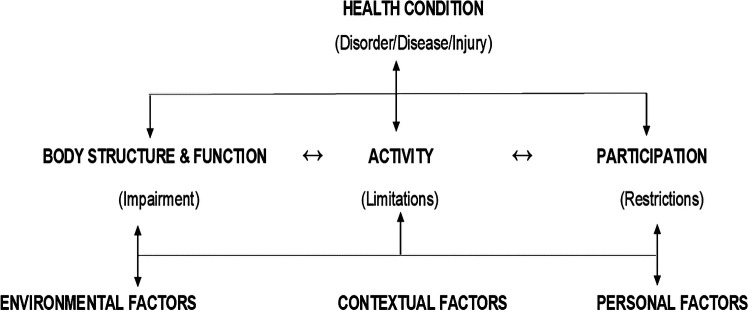
ICF framework (adapted from world health organization)^6^.

Students in the second year of study are introduced to the international classification of function, disability and health (ICF) framework (Figure 1) (21). The framework emphasizes a biopsychosocial approach to rehabilitation. It highlights the person's health condition and emphasizes the interactions between the person's participatory restrictions, activity limitations, and body impairments with the contextual factors (environmental and personal). The ICF underpins the interventions provided by physiotherapists and other rehabilitation professionals such as audiologists, speech and language therapists, and occupational therapists.

Advocacy and inclusion of the marginalized population remains a focus of the curriculum. Additional concepts that are taught in the second year are the determinants of health (22). Students are taught the multi-level framework of applying the determinants of health, using the self-health assessment students are expected to assess their own health status at individual and household level. Understanding health and society is central to the second-year curriculum as this further prepares students for clinical and community placement at the third-year level.

To apply diversity, students are required to identify an under-resourced and marginalized community from an urban and a rural community of South Africa. They then must discuss possible health issues focusing on inclusion and access to rehabilitation services for the identified community. This learning allows students to identify their rehabilitative role as physiotherapy students and apply learned concepts to a real-life community setting.

The students are also put in a simulation situation where they get to understand social exclusion. They are split into two groups, where one group assumes the role of a physically disabled person and the other group assumes the role of a health worker or government official in charge of policy implementation. The disabled student must navigate the university premises in a wheelchair, crutches, or blindfolded. The role of the health worker is to conduct a subjective assessment of the disabled person to get an understanding of their daily challenges. The government official communicates with the health worker to discuss strategies to implement structural and social policies that enable inclusion. This highlights the need for persons with disabilities to be included in the discussions of policies that affect them, further highlighting the concept of “for the people, by the people”.

### Theory to practice: third and fourth year of study

In the third and fourth years of study, students are placed in urban and rural community health centres, community health clinics or rural district hospitals to fulfil a set of clinical objectives. The students are required to engage with relevant stakeholders to develop needs-based community projects which address the issues of diversity, inclusion, and equity as listed in Table 1.

To understand the rehabilitation needs and ensure sustainability of programmes, students are expected to keep and update a community file. The file contains all information on community projects performed by previous students and includes recommendations for project sustainability. Students engage with rehabilitation health workers, clinic staff such as nurses, community leaders, community structures such as schools, and they also perform a transect walk of the community to identify the determinants of health and issues of accessibility within the community.

To bridge the gap of accessibility to the health facility, physiotherapy students perform a home visit (home-based rehabilitation) to an identified community member with a disability or physical limitation. The aim of this home-based rehabilitation is to assess and intervene an individual's functional capacity in their own home when that person would otherwise not be able to access the local health facility. Community reintegration and caregiver strain relief forms part of the focus for home-based rehabilitation.

Patients who are identified as experiencing common disabling impairments receive group rehabilitation and social support groups are created for patients and caregivers of people with disabilities. The physiotherapy students are expected to identify a community structure such as a school, disability centre, or a community social club and assess the rehabilitative needs of this structure. Interventions such as health education and health promotion are applied based on the identified need. Students also get to engage with local community members which further deepens their understanding of the determinants of health in the community. Students are then able to provide rehabilitation services to those who are marginalised, and further involve other health workers and external stakeholders. To inform the community about rehabilitation services, students provide education at health facilities waiting areas. Students also promote physiotherapy at schools within the community to encourage learners from rural backgrounds to pursue a career in physiotherapy.

Access to rehabilitation services was further worsened by the COVID 19 pandemic (23) when national lockdown was implemented (24). Due to the country's movement restriction, fourth-year students were placed at urban primary health care facilities for their public health and community physiotherapy block. An example of a project that was conducted during the COVID 19 pandemic is illustrated below:

**Case study 1 COVID 19 pandemic services rendered by physiotherapy students.**

Students conducted community projects ranging from health education, health promotion, health screening, home-based rehabilitation, and occupational health. The rehabilitative role was highlighted during house-to-house visits to patients who could not access health facilities due to personal disability limitations, inability to afford transport to the point of care, over-crowding in community clinics and various COVID-19 protocols such as social distancing. Outreach services were conducted with community health promotors to expand access and inclusion to nongovernment organizations for the elderly and people with disabilities. The students provided in-service training on mental health, COVID-19 screening, health and wellbeing of nurses, health promotors, and community health workers. Students advocated and raised funds for resources such as cloth face masks and hand sanitizers for different community organizations.

Students compiled learning portfolios and kept a reflective diary on their experiences during clinical blocks. From the student diary reflections, it was evident that students felt proud to have made a difference and be active agents of change. Students expressed feelings of anxiety, stress, frustration, and fear during the service-learning process. However, the process developed resilience in the students.

As a core function of public health, health promotion allows for the awareness of health issues by supporting individuals to cope with and address various health challenges. Students in the third and fourth year of study conduct health education sessions to promote health knowledge and self-management of at-risk populations. During these sessions, students must account for various factors such as language barriers, level of literacy of the audience and provide specific, evidence-based community-centred information all whilst demonstrating respect, professionalism, adaptability to the setting that they are in.

Through the fourth-year curriculum, public health laws are taught to help students understand the “bigger picture”, whereby political marginalization, discrimination and inequitable access to health services are experienced by persons with disabilities resulting in poorer health outcomes (25). Students are therefore aware of policies guiding health and rehabilitation and can advocate and mediate on behalf of their patients and/or the community they are placed in.

## Conclusion

Physiotherapy training should not only include clinical skills but must incorporate fostering the students' ability to promote health and address issues of access, equity, diversity, and inclusion. In the context of challenges faced by people with disabilities as well as those at risk of developing disabilities, students can be agents of change and drivers of universal health coverage as they reach marginalized communities through outreach activities related to rehabilitation. We conclude by offering a reflection of our experience in teaching students to provide equitable access to physiotherapy services for marginalized communities.

### Author 1

Getting into physiotherapy as a career was never part of the plan. It became a destiny and a personal agenda when I got exposed to public health and community physiotherapy. Since my involvement in training physiotherapy students and working with vulnerable communities, I have grown to feel like a useful member of society. I have learnt that people living in low-resource communities are very knowledgeable about health topics and have a desire to learn more, but the system does not always provide the opportunity. People are always looking to make use of the system built for them to either promote, prevent, or rehabilitate themselves but this is constantly denied due to the inaccessibility of the system. A moment in my public health and physiotherapy training career that I will forever remember was when I conducted a home visit with third year students to a client who survived a stroke, was bed-bound, and also blind. She had not been outside her bedroom for eight years since the diagnosis of stroke due to fear and lacked the motivation and reassurance of getting into a wheelchair and going outside. When the students confidently transferred her to the wheelchair and persuasively got her out of her front door into the sun she cried for minutes and repeatedly said to the students it is the best healing she has ever received as a stroke survivor.

### Author 2

My experience as a clinical educator has allowed me to observe students from various backgrounds entering communities which contrast their own. During health education talks, students often provide advice on nutrition and exercise to patients in the waiting area of the Community Health Centre. Students were often aware of the setting and tailored their education to suit the diverse needs of the community. For example, students may explain healthy foods to eat during pregnancy in terms of what is available, accessible and cost-effective within the community. Elements of inclusion, equity and diversity are often noted when health education is specific and relatable to the community. However, some students often fail to adapt this to the community and do not consider the diversity of the audience to whom they were speaking. This is noted when they mention physical activities such as swimming, accessing a gym or using exercise equipment which many individuals in the community would not have access to.

Students are encouraged to include elements of diversity, equity and inclusion in every step of their clinical practice, patient engagement and professional lives. However, while these elements may be included well in theoretical training I have observed that some students experience a “culture shock” when placed in an environment which contrasts significantly to their personal lives. Acknowledgement that some students adapt to the circumstances and incorporate the elements is not enough to institute change. We need to garner an understanding of the reasons why some students can understand the importance of equity, inclusion and diversity and include it in their clinical practice and why some cannot. This will assist in tailoring curricular to produce healthcare professionals who note these elements as core principles in patients care.

### Author 3

In my experience as a clinical educator, I have engaged with students from many walks of life. I have always believed healthcare professionals have a “calling”, that no matter how hard you try to run away from it, the profession always chooses you. My training as an undergraduate student will always be a reminder that healthcare is complex and multifaceted, a season that will require you to plant yourself in unfamiliar territory so you can anchor yourself where growth potentially exists. Thereafter as a qualified healthcare professional, it is up to you to water yourself in other seasons of your career for continued growth. I will always encourage the students that cross my path to submerse themselves in the unknown, to get familiar with practicing reflection of self, but more than that to treat all patients with dignity and respect regardless of their socioeconomic status or cultural background, to approach every patient with empathy and compassion as if it were their own family member. A moment that stood out so vividly for me and pushed my passion for students' training was a home visit whereby students displayed attitude and disinterest towards the patient all because he had not bath yet and his home was untidy. I was disheartened and appalled by their attitude and body language, and quickly had to remind them that just because someone looks or smells different does not make them less of a human. That regardless of their home environment they still need our services, and we have to treat each patient with respect and dignity.

### Author 4

I have taught public health and community physiotherapy as well as supervised physiotherapy students for ten years. I believe in holistic person-centred care where patients and community members are central to healthcare. Over the years I have observed that exposing students to urban and rural settings helps the students appreciate the diverse social context health care is delivered in. Some settings where students conduct their activities highlight the inequalities our country faces such as differences in household income, personal safety experiences, access to adequate housing, sanitation and clean water. Such experiences help students gain a better understanding of the challenges patients face and the need for social justice and efforts to address inequity. Two experiences stand out for me: I went with 3rd year students to location × for a health intervention for people with strokes. The hall was full of immigrants, young and old who needed health information on how to manage their stroke. The students conducted a health talk in English but needed to adapt and make the message accessible to the French speaking audience. My second experience is based on a home visit conducted by 4th year students in a rural setting where the students assessed a teenage girl with a spinal cord injury and her house environment. The house where the patient lived was not accessible and the father had to carry her outside the house. The students partnered with the local business community and the local businesses donated material to build a ramp. Such interventions highlight responsive care that ensures access to health care services and addressing inequity.

## Data Availability

The original contributions presented in the study are included in the article, further inquiries can be directed to the corresponding author.
